# Hydrophilic Mineral Coating of Membrane Substrate for Reducing Internal Concentration Polarization (ICP) in Forward Osmosis

**DOI:** 10.1038/srep19593

**Published:** 2016-01-22

**Authors:** Qing Liu, Jingguo Li, Zhengzhong Zhou, Jianping Xie, Jim Yang Lee

**Affiliations:** 1 Department of Chemical & Biomolecular Engineering, National University of Singapore, 10 Kent Ridge Crescent, Singapore, 119260

## Abstract

Internal concentration polarization (ICP) is a major issue in forward osmosis (FO) as it can significantly reduce the water flux in FO operations. It is known that a hydrophilic substrate and a smaller membrane structure parameter (S) are effective against ICP. This paper reports the development of a thin film composite (TFC) FO membrane with a hydrophilic mineral (CaCO_3_)-coated polyethersulfone (PES)-based substrate. The CaCO_3_ coating was applied continuously and uniformly on the membrane pore surfaces throughout the TFC substrate. Due to the intrinsic hydrophilicity of the CaCO_3_ coating, the substrate hydrophilicity was significantly increased and the membrane S parameter was reduced to as low as the current best of cellulose-based membranes but without the mechanical fragility of the latter. As a result, the ICP of the TFC-FO membrane could be significantly reduced to yield a remarkable increase in water flux without the loss of membrane selectivity.

Forward osmosis (FO) is a membrane process which draws water from the feed solution (low osmotic pressure) to the draw solution (high osmotic pressure) solely based on the osmotic pressure difference as the driving force. Compared with conventional pressure driven membrane processes such as nanofiltration (NF) and reverse osmosis (RO), FO has the benefits of a lower fouling tendency, high water recovery and mild operating conditions. Since water can be transferred without external forces, FO is particularly suited for applications where the regeneration of the draw solution is not needed, such as irrigation and hydration bags. In addition, with continued R&D on the regeneration methods for draw solution, seawater desalination and wastewater treatment by FO may also become technologically possible^1^,^2^,^3^,^4^,^5^,^6^,^7^,^8^.

The main hurdles in the development of FO processes are the dearth of easily regenerable draw solution and the limited selection of commercial FO membranes^4^,^9^,^10^,^11^,^12^. While cellulose- based flat sheet membranes are the most common commercially available FO membranes, they have relatively low salt rejection and strong propensity for hydrolytic degradation and biodegradation^9^,^10^. On the other hand, thin film composite (TFC) membranes have dominated the RO market because of their high water flux and salt rejection properties, as well as mechanical and chemical stability. Hence it is only natural that the TFC membranes have also been applied to FO processes^9^,^10^,^11^,^12^,^13^,^14^,^15^,^16^,^17^,^18^,^19^,^20^,^21^,^22^. A typical TFC-FO membrane is composed of two layers: a porous substrate formed by phase inversion for mechanical support; and a thin permselective polyamide (PA) layer formed by the interfacial polymerization (IP) between an amine such as m-phenylenediamine (MPD) and an acid chloride such as 1,3,5-benzenetricarbonyl trichloride (TMC). This design allows the substrate and the selective layer to be independently customized to meet various functional requirements.

Due to the different water transport mechanisms in RO and FO, the conventional TFC-RO membranes show rather poor water permeability in FO processes^9^,^10^,^11^. The TFC-FO membranes therefore have to be designed differently from the RO membranes, especially with regard to the support layer (substrate). A dense and thick substrate is necessary for a TFC-RO membrane to withstand the high hydraulic pressure in RO operations. The same thick substrate in pressure-free FO operations would only increase the internal concentration polarization (ICP) in the membrane substrate and consequently reduce the water flux^23^,^24^,^25^,^26^,^27^. ICP is caused by the combined effect of draw solution dilution by water emigrated from the feed into the support layer; and by the resistance to draw solute diffusion from the bulk of the draw solution to the substrate. The effective osmotic pressure difference across the selective layer can be substantially lower than the osmotic pressure difference of the feed and draw solutions. Unlike external concentration polarization, ICP cannot be mitigated by changing hydrodynamic conditions such as increasing flow rate or turbulence. For a TFC-FO membrane, while the selective layer is central to salt rejection and water flux, the substrate ICP can also affect the water flux in a significant way^4^.

It is generally known that a small membrane substrate structure parameter (S = thickness × tortuosity/porosity) is beneficial to ICP reduction^19^,^20^,^21^,^28^,^29^. Thus the substrate should be thin and porous with extensively interconnected pores to facilitate solute diffusion from the draw solution to the selective layer, and yet has adequate mechanical, chemical and thermal stability to withstand industrial operations. Hydrophilicity and wettability modifications of the substrate have been the main methods of ICP mitigation^10^,^11^,^12^,^13^,^17^,^18^,^19^,^20^. It is assumed that a hydrophilic substrate not only improves the transport of water and solute molecules, but also enhances the substrate wettability. Effective porosity can be increased and effective tortuosity decreased by reducing air entrapment in the membrane pores; and a smaller S parameter results. The faster transport of water and solute molecules together with a smaller S parameter contributes to a lower ICP and higher water flux. Hence the hydrophilization of TFC-FO membrane substrate is an active research area within the membrane community.

One of the most common hydrophilization methods is the incorporation of a hydrophilic polymer (e.g. sulfonated polymer) into the hydrophobic membrane substrate^12^,^13^,^17^. The hydrophilized substrate often shows weakened mechanical properties due to the increase in swelling by water. What’s more, the hydrophilic polymer tends to elute, which is hard to recover. Physical treatment after membrane fabrication such as oxygen plasma induced hydrophilicity has also been used but the membrane mechanical properties are again compromised. Inorganic additives, with their inherently good mechanical properties, have also been used for hydrophilicity improvements. For example, zeolite and titanium oxide particles were introduced as a dispersed phase to increase membrane hydrophilicity^19^,^21^. This often requires the fillers to be on the nanoscale and the use of a linker. The uniform distribution of nanosized fillers is a challenge and places a limit on the effectiveness of hydrophilicity improvements through filler addition.

Hence the design of a FO membrane should begin with the selection of a polymer with good membrane forming properties as well as good mechanical, chemical (including hydrolytic and biological) stability. Polyethersulfone (PES) fits many of these descriptions except that it is also highly hydrophobic and as such is not suitable for FO application without modifications. The hydrophilization method should be free from the previously described deficiencies. Inspired by nature’s bio-mineralization process and the intrinsic hydrophilicity of minerals, inorganic mineral coating could be an alternative means to impart hydrophilicity to a PES substrate without loss of other functional properties. Biomineralization is the formation of a stable organic-inorganic hybrid by the interaction between specific organic and inorganic ions. CaCO_3_ is a good biomineral which also has high hydrophilicity due to ionic and hydrogen bonding with water molecules. In order to initiate the nucleation of CaCO_3_ in PES, a common polyanionic macromolecule such as polyacrylic acid (PAA) was introduced to the PES substrate. Through the electrostatic interaction between the carboxylate groups of PAA and Ca^2+^, the coverage and continuity of the CaCO_3_ coating in PES could be progressively built up to improve the hydrophilicity of the PES substrate without the trade off in its mechanical strength.

Herein, we apply a previously reported alternative soaking process (ASP)^30^,^31^ to produce TFC-FO membranes with a CaCO_3_-coated PES substrate. The successful hydrophilization throughout the PES substrate was evidenced by the substantial decrease of water contact angle, and a very small S value comparable to the lowest value reported to date (which was cellulose-based membrane) but with greater mechanical stability nature^22^. As a result, the water flux not only increased after the membrane modification, but also surpassed many of the FO membranes in current research especially when operating in the AL-FS mode (where the active layer faces the feed solution). The ASP has been previously used to improve the hydrophilicity of microfiltration membranes^30^,^31^, which are single-layered membranes formed by the phase inversion method. The application of ASP to the heterogeneously structured TFC membranes (consisting of a substrate and a selective IP layer) is more challenging - it is necessary that the CaCO_3_ coating is applied only to the substrate so as not affect the performance of the membrane selective layer. This study therefore provides a possible solution to the ICP problem in FO operations without any tradeoff effect. The generality of the method suggests that it may also be applicable to other membrane substrate materials.

## Results

### Preparation of CaCO_3_-coated TFC-FO membrane

**Figure 1:**
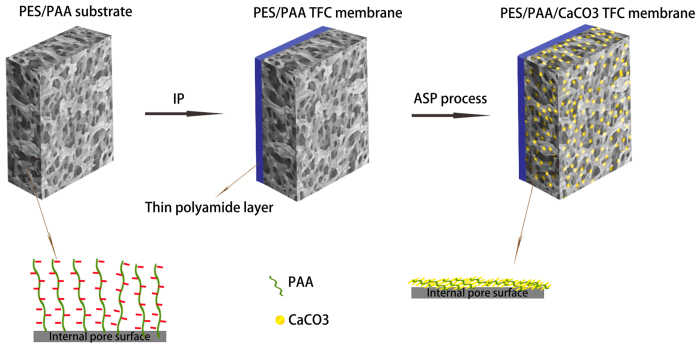
Schematic illustration of the preparation of CaCO_3_-coated TFC membranes.

Figure 1 shows the three main steps in the preparation of CaCO_3_-coated TFC-FO membranes. First, a negatively charged substrate was formed by the standard phase inversion method using a PAA-blended PES dope. A selective layer was then formed on the top face of the substrate by interfacial polymerization. Finally ASP was used to coat the substrate interior surface with CaCO_3_ by first adsorbing Ca^2+^ ions onto the membrane pore surfaces by electrostatic forces, followed by reaction with CO_3_^2−^ ions to form the hydrophilic mineral CaCO_3_.

PAA is commonly used as an additive in membrane modifications to impart hydrophilicity. In this study, however, the main objective of PAA addition was to enable chemical interactability between the PES substrate and Ca^2+^. In comparison with other hydrophilic polymers such as polyvinyl alcohol (PVA), the negative charge developed after PAA dissociation in water could chemically bond Ca^+^ through electrostatic forces to initiate the CaCO_3_ coating^32^,^33^,^34^,^35^. This property is derived from an abundance of carboxylic groups in PAA with pKa (4.75) lower than water (7.0). The casting dope in this study was prepared by a standard method where clear PES and PAA dopes were prepared separately and mixed in different proportions. It was reported that a semi-interpenetrating polymer network could be formed with this method^34^.

**Figure 2:**
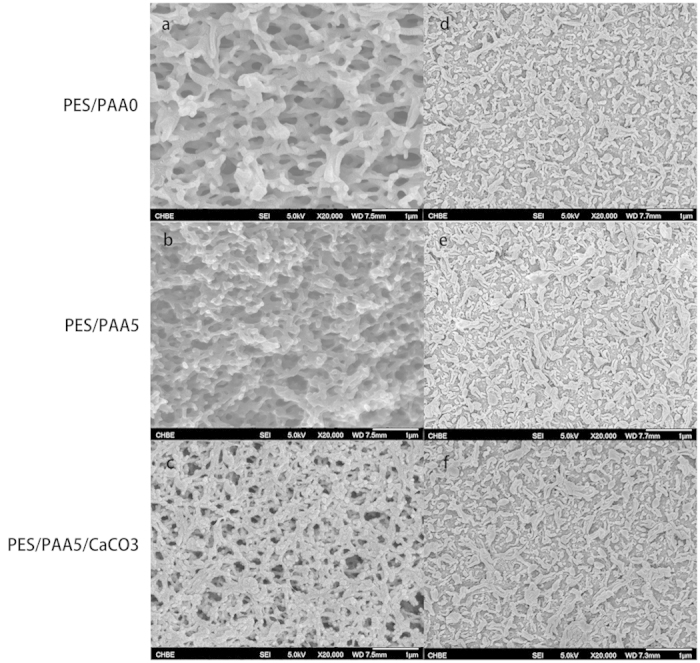
FESEM images of the morphology of the substrate (**a**–**c**) and the selective layer (**d**–**f**).

The selective layers on the top surfaces of PES/PAA0, PES/PAA5 and PES/PAA5/CaCO_3_ membranes were all morphologically alike with a typical ridge and valley appearance (Fig. 2d–f). This is an encouraging sign which suggests that the properties of the skin layer, which determine the membrane selectivity, were likely not affected by the incorporation of PAA or the ASP treatment. This is to be expected from the lack of chemical affinity between the PA layer and Ca^2+^. In addition, the membranes were rinsed in water after each soaking step to remove the physisorbed Ca^2+^ ions on any non-interacting surfaces. Hence no CaCO_3_ crystallites were formed on the selective layer.

Contrary to the sameness of the selective layer, there were significant changes in the PES-based membrane substrate after PAA modification and after ASP treatment (Fig. 2a–c). The polymer matrix of the PES/PAA5 membrane was roughened by the ASP treatment, which may be used to suggest successful CaCO_3_ coating and an indirect proof for the presence of PAA which enabled the deposition. It can be seen that the coating was quite conformal, retaining many of the architectural features of the porous membrane substrate. Energy dispersive X-Ray spectroscopy (EDX) confirmed the presence of Ca and its uniform distribution throughout the entire membrane substrate (Figure S1).

**Figure 3:**
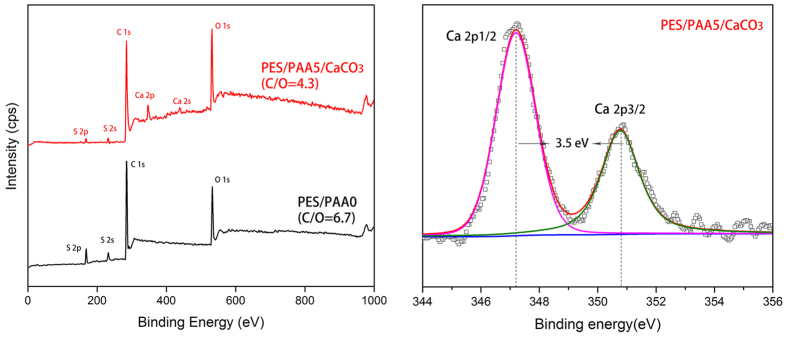
XPS wide scan and Ca 2p core level spectrum of PES/PAA0 and PES/PAA5/CaCO_3_ membranes.

The presence of CaCO_3_ in the membranes was independently confirmed by XPS. Figure 3 shows the wide scan XPS spectra and the Ca core level spectra of the bottom surfaces of PES/PAA0 and PES/PAA/CaCO_3_ membranes. The extra peaks in the wide scan spectrum of the PES/PAA/CaCO_3_ membrane correspond well with the Ca element. The two peaks in the Ca 2p region with binding energies of 346.8eV and 350.3eV are in perfect agreement with the characteristics of Ca 2p^1/2^ and Ca 2p^3/2^. In addition, the wide-scan XPS spectrum of PES/PAA/CaCO_3_ also revealed an increase in oxygen content and a decrease in C/O ratio (=4.3) relative to PES/PAA0 (=6.7) (Fig. 3). This is consistent with the consequence of PAA blending and CaCO_3_ deposition. As shown in Figure S2, the C 1s peak could be deconvoluted into several component peaks representing different chemical environments. The two peaks at ~284.6eV and 268.2eV in the C 1s spectrum of the PES/PAA0 membrane could be attributed to C-H and C-O carbons respectively. An additional peak at 288.5 eV corresponding to the O-C=O carbon was found in the spectrum of the PES/PAA/CaCO_3_ membrane. This is further proof for the presence of CaCO_3_ in the membrane substrate. Except for XPS, XRD analysis of the coated membrane also indicated the characteristic diffraction of the (−112) planes of CaCO_3_ at 2θ of 29.7° (Figure S3). Inductively coupled plasma optical emission spectrometry (ICP-OES) of the PES/PAA/CaCO_3_ membrane indicated a Ca content amounting to 2.34% wt. of the membrane.

### Effects of mineral coating on membrane properties

#### Membrane hydrophilicity

**Figure 4:**
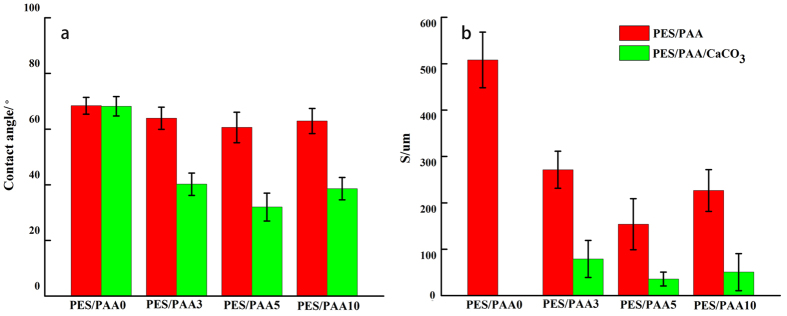
Contact angle (**a**) and structure parameter (**b**) of the membranes.

Figure 4 shows the changes in the membrane surface properties before and after CaCO_3_ coating. The changes in membrane substrate hydrophilicity were evaluated by the standard method of water contact angle measurements. The membrane substrate water contact angle was noticeably reduced after CaCO_3_ coating, suggesting that CaCO_3_ was able to significantly increase membrane hydrophilicity. Previous research^36^ has shown that CaCO_3_ has a high surface free energy and forms hydration layer easily through ionic and hydrogen bonding. Since the CaCO_3_ coating was contiguous throughout the membrane substrate, the hydrophilicity improvement was not limited to the membrane exterior surface but also the interior surfaces of the membrane substrate.

Blending PES with PAA is known to increase membrane hydrophilicity due to the carboxylic groups of incorporated PAA^33^ but such contribution was relatively minor in this study. This is because the hydration ability of the carboxylate groups of PAA is far lower than that of CaCO_3_. Similar to the findings of Yu *et al.* 36, an optimum PAA concentration existed for which the water contact angle was the smallest. In this study that composition corresponded to the PES/PAA5 membrane. Even with a dope preparation method which promotes polymer entanglement, hydrophilicity could not be increased beyond a threshold PAA loading of 5 vol% since excess PAA molecules could be eluted out of the membrane due to their water solubility and hence contributed little to the hydrophilicity of the membrane substrate.

The trend in contact angle changes after CaCO_3_ coating was similar to the trend before CaCO_3_ coating. i.e. the lowest contact angle was again registered by the PES/PAA5/CaCO_3_ membrane. The main function of PAA blending in this study was to introduce negative surface charge and to provide the sites for CaCO_3_ nucleation. Thus the more PAA that could be retained in the substrate, the more carboxylate groups would be available for electrostatic binding with Ca^2+^. A larger number of CaCO_3_ crystallites could then be formed and deposited on the pore surface. In order to confirm the function of PAA in the preparation of the mineral coated membranes, the ASP treatment was also applied to the PES/PAA0 membrane (i.e. a neat PES membrane). As per our expectation the PES/PAA0/CaCO_3_ membrane did not show any decrease in the contact angle (ICP-OES also did not detect any Ca), indicating the blending with PAA is essential to the success of the CaCO_3_ coating.

It should be noted that the CaCO_3_ hydrophilization of membrane substrate did not bring about any negative effect on the membrane mechanical properties. There was some reduction of the membrane mechanical properties after blending with PAA (Table S1). This was caused by hydrophilic polymer swelling and is common to the use of hydrophilic polymer to improve membrane hydrophilicity^12^. Interestingly mechanical properties were improved after the ASP treatment. Indeed the mechanical strength was even higher than the control (pristine) PES membrane. This could be credited to the strengthening of membrane substrate strengthening by a conformal CaCO_3_ coating.

#### Transport properties and membrane structure parameter (S)

Water permeability (A) and salt (NaCl) rejection (Rs) of the TFC membranes measured in the RO mode are shown in Figure S4. It can be seen that the addition of PAA has led to a slight decrease in A. According to prevailing views, PAA can affect the membrane performance through two opposite effects: an increase in hydrophilicity which improves the water flux, and a PAA-swelling induced shrinkage which blocks the membrane pores and reduces the water flux^33^. The actual water flux is the balance between these two opposing effects and is dependent on the extent of PAA addition. The effects of substrate hydrophilicity and wettability are less important in the RO mode (relative to the FO mode) where water is primarily “pushed through” by the externally applied hydraulic pressure. Other than hydrophilicity improvement, CaCO_3_ coating may cause the collapse of the PAA chains to create extra space for the diffusion of water and salt in the substrate^30^,^31^. The combined effect resulted in the CaCO_3_ coated membrane showing the comparable intrinsic water permeability as the pristine PES membrane (PES/PAA0) in the RO mode. On the other hand, the salt rejection properties of all membranes were quite similar and were above 90%. This is indication that the selective layer remained in a good condition after the ASP treatment.

The structure parameter S is often used to estimate the severity of ICP in FO processes. A small structure parameter is desirable for the FO membranes. Clearly, the S parameter of the membrane was all lower after coating with CaCO_3_ (Fig. 4). The decrease should be attributed mostly to the increase in hydrophilicity and wettability. Unlike in RO, a FO membrane must be fully wetted in order to support efficient water transport and to maintain the continuity of the water phase in the substrate without the assistance of an external hydraulic pressure. With the enhancement of hydrophilicity and wettability, the lowest S parameter (35.7 um) was obtained with the PES/PAA5/CaCO_3_ membrane. This value of the S parameter is comparable to the lowest S value reported by Ong *et al.* (31.9 um)^22^. In that study, the low S value was obtained with the help of a very thin cellulose ester substrate and hence mechanical strength can be an issue. In addition, unlike the PES-based membranes, cellulose-based membranes are very susceptible to hydrolytic and biological decomposition. The S parameters of all three CaCO_3_-coated membranes were all below 100 um, which are lower than most TFC membranes in the literature. Since a smaller S value is indicative of a smaller ICP effect, a higher water flux is expected of these CaCO_3_-coated membranes in the FO operation.

### Effect of mineral coating on membranes FO performance

**Figure 5:**
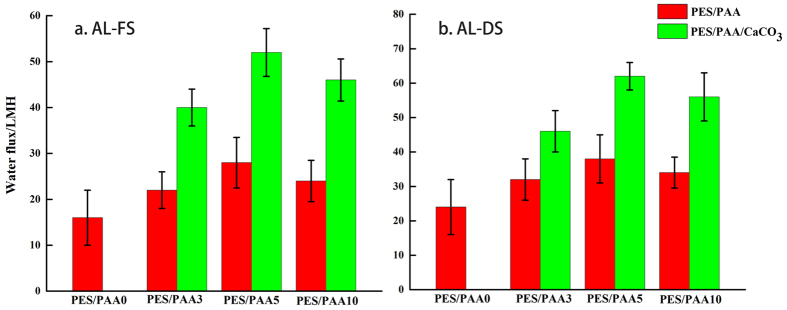
FO water flux of membranes in (**a**) AL-FS orientation and (**b**) AL-DS orientation.

Figures 5 and S5 shows the water flux and the reverse salt flux of the membranes measured in a standard cross-flow FO set-up. The trends of water flux and salt flux were similar in both orientations. The higher water flux amidst a slight increase in the salt flux in the AL-DS mode is within expectation due to the inherently lower ICP in this FO orientation. It can be seen that the variation of water flux with PAA loading followed the hydrophilicity trend. Consequently among the three PAA blended membranes, the PES/PAA5 membrane with the highest hydrophilicity (lowest contact angle and S value) also exhibited the highest water flux. This is suggestion that the positive effect of hydrophilicity improvement had more than compensated the negative effect of pore shrinkage and blockage caused by PAA swelling.

Furthermore, the CaCO_3_- coated membranes showed a significant improvement of the water flux. The highest water flux in both AL-FS and AL-DS modes was from the PES/PAA5/CaCO_3_ membrane, at about 52 LMH and 62LMH respectively. The water flux from the AL-FS mode was about 3.25 times of that of the unmodified PES membrane, and 1.85 times of that of the corresponding PAA blended membrane. The enhancement was due to the substantial ICP reduction resulting from the enhanced water and salt transport. AL-FS is the preferred orientation in practice because of more manageable membrane fouling^4^. In this study, the greatly enhanced water flux in the AL-FS mode also surpassed most FO membranes in the published literature (Table 1)^9^,^12^,^13^,^17^,^18^,^19^,^21^,^22^,^28^,^37^.

**Figure 6:**
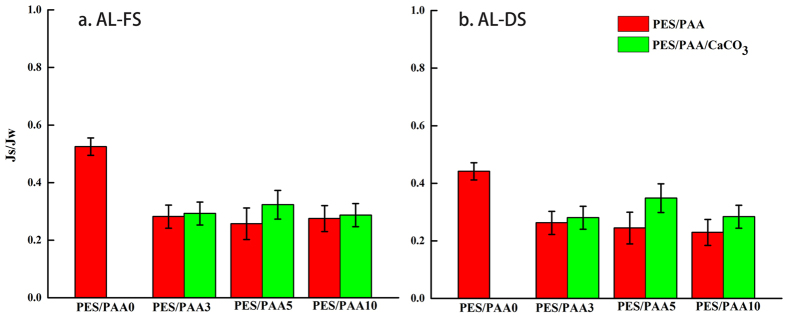
Js/Jw values of membranes in both (**a**) AL-FS (**b**) AL-DS orientations.

As per general practice, the selectivity of the PA layer was also evaluated by the ratio of the reverse solute flux to the water flux, Js/Jw, in both AL-DS and AL-FS modes, for all of the membranes (Fig. 6). In general, a low Js/Jw value is an indication of good membrane selectivity in rejecting solute relative to water. In this study, the Js/Jw values of all the CaCO_3_-coated membranes in both AL-DS and AL-FS modes were smaller than the PES/PAA0 membranes, suggesting improved selectivity of the PA skin layer after CaCO_3_ modification. This could be due to the improvement of substrate hydrophilicity and substrate wettability which benefited water transport more than salt transport. In conclusion, with a more hydrophilic substrate the water flux of the FO membranes in this work could be considerably improved without noticeable penalty in selectivity.

It should be mentioned that CaCO_3_ is slightly soluble in water. Its release in water causing the gradual loss of hydrophilicity could lead to a water flux decrease. An extended FO performance test was therefore carried out in the AL-DS mode to assess the extent of Ca dissolution. The feed solution was replaced with DI water every 12 hours, and the Ca content in the feed solution Ca was measured with ICP-OES. The results (Figure S6) showed that the Ca release rate was progressively reduced, and in 72 hours, about 38% of the initial Ca content in the membrane was lost. This rate of Ca release also led to about 30% decrease in the water flux (Figure S7). The remaining water flux was however still higher than PES and PES/PAA membranes without the Ca^2+^ modification. The Ca loss could also be easily replenished by another round of ASP, with over 95% recovery of the initial water flux. This was due to the good retention of PAA, the nucleation sites for CaCO_3_, in the membrane substrate. Thus it can be seen that besides the above advantages, the CaCO_3_ coating also possesses added advantages of 1) replenishability and 2) inhibition of PAA leakage from the PES/PAA membranes^38^. The two effects are intertwined and hence even if CaCO_3_ elution occurs after extended use, the re-exposed PAA would allow a replacement CaCO_3_ to be formed by repeating the ASP treatment.

## Discussion

CaCO_3_-coated FO TFC membranes were used in this study to reduce the ICP in FO operations. By blending PES with PAA, a uniform CaCO_3_ coating could be deposited uniformly throughout the interior pore surfaces of the blended membrane substrate. This CaCO_3_ coating increased the hydrophilicity of FO-TFC membranes substantially, as shown by the substantial reduction of the water contact angle. As a result of the increase in hydrophilicity, the coated membranes provided significantly lower S parameters, with the lowest one comparable to the best that is possible with common cellulose-based membranes but with much greater stability. Consequently the FO water flux could be significantly increased without the loss of membrane selectivity. This study therefore demonstrates that CaCO_3_ coating is effective for migrating ICP in FO processes.

## Methods

### Materials

Radel ^®^ A polyethersulfone (PES) from Solvay Advanced Polymers; N-methyl-2-pyrrolodinone (NMP, >99.5%), polyethylene glycol 400 (PEG, Mn = 400 g/mol) and PAA (Mn = 45,000) from Merck; MPD (>99%), TMC (98%), hexane, calcium chloride, sodium carbonate and sodium chloride from Sigma-Aldrich, were all used as received. De-ionized water was produced by a Milli-Q ultrapure water system (Millipore, USA).

### Membrane preparation

The PES membrane substrate was prepared by the Loeb-Sourirajan dry-wet phase inversion method. In brief, PES polymer was first dried in vacuum overnight for moisture removal. It was then mixed with PEG-400, NMP and water to form Dope 1. Separately PAA was dissolved in NMP to form Dope 2. Table 1 shows the detailed compositions of the two Dope solutions. The two solutions were mixed in different proportions to form the various casting solutions in Table 2. The rested casting solution after overnight degassing was cast onto a glass plate with a 100 μm casting knife. The as-cast membrane was immediately immersed in a water coagulation bath at room temperature and kept there for 24 h to ensure complete precipitation.

A PA selective layer was formed on the membrane top face by the following procedure: a membrane substrate was immersed in a 2 wt% MPD aqueous solution for 1 min. The excess MPD solution was absorbed by a filter paper. This was followed by contacting the same membrane surface with a 0.1 wt% TMC solution in n-hexane for 30 s. The TFC membrane fabricated as such was air-dried for 5 min and then stored in deionized water.

The formation of a CaCO_3_ coating in the membrane was based on ASP. A TFC membrane was consecutively soaked in CaCl_2_ aqueous solution (200 mmol/L), DI water, Na_2_CO_3_ aqueous solution (200 mmol/L), and DI water for 1min each. This soaking sequence was then repeated for three cycles (the “optimal” number of cycles in this work after repeated trials). The membranes after ASP treatment were stored in deionized water before use.

### Membrane characterization and performance measurements

Membrane morphology was examined by field-emission scanning electron microscopy (FESEM) on a JSM 6700F (JEOL, Japan) microscope operating at 5 kV accelerating voltage. The membrane samples for FESEM examination were first dried in a ModulyoD freeze drier (Thermo Electron Corporation, USA), followed by cryo-fracturing in liquid nitrogen and Pt surface coating in a JFC 1300 auto fine coater (JEOL, Japan).

The membrane chemical compositions were measured by X-ray photoelectron spectroscopy (XPS) on a Kratos AXIS Ultra HSA spectrometer with a monochromatic Al Kα source (hν = 1486.6 eV) operating at 15 kV and 5 mA.

The presence of CaCO_3_ was determined by X-ray diffraction (XRD) using a M18XHF-SRA (Mac Science) diffractometer and a Cu Kα radiation source (k = 1.5406 Å) at 40 kV and 300 mA (12 kW). The diffraction between 2θ of 20° and 80° was scanned at 2° min^−1^.

Contact angle measurements were carried out in water at room temperature, using a Contact Angle Goniometer (Rame Hart) and Milli-Q deionized probe liquid to measure the hydrophilicity of the membrane surface.

The mechanical properties of membrane substrates were measured by using an Instron 5542 tensile testing equipment. The flat sheet membranes were cut into stripes with 5 mm width and clamped at the both ends with an initial gauge length of 30 mm and a testing rate of 10 mm/min. At least five stripes were tested for each casting condition to obtain the average values of tensile stress, Young’s modulus and elongation at break of the membranes.

A dead end stainless steel cell was used for the measurements of the membrane intrinsic transport properties^9^,^12^. Water permeability (A), salt permeability (B) and salt rejection (R) of the fabricated membranes were measured in the RO mode. The effective membrane area in the RO filtration equipment was 9.62 cm^2^. Water permeability A was calculated from the pure water permeation flux under a transmembrane pressure of 3 bar by equation 1 where J_v_ is the volumetric water flux and ΔP is the applied hydraulic pressure.


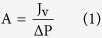


Salt rejection (R) was calculated from equation 2, where C_p_ was _p_ermeated salt concentration and C_f_ was feed salt concentration (200 ppm NaCl). The salt concentrations were measured indirectly from conductivity readings on a conductivity meter (Lab 960, SI analytics GmbH, Germany).


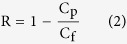


Salt permeability coefficient B was calculated according to equation 3, which is based on the solution-diffusion theory. Δπ is the osmotic pressure across the membrane^39^.


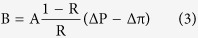


According to the classical ICP model developed by Loeb *et al.*, FO water flux can be calculated from equations 4 and 5 for the AL-FS and AL-DS (selective layer facing the draw solution) orientations respectively^40^,^41^.


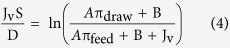



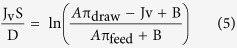


In the above equations π_draw_ and π_feed_ are respectively the osmotic pressure of the draw solution and the feed solution, and D is the salt diffusion coefficient.

### FO performance of TFC membranes

A lab-scale cocurrent-flow filtration unit was used for the FO measurements as in previous research^9^,^12^,^17^,^22^,^23^. The permeation cell was a standard plate-and-frame design with a rectangular channel (2.0 cm in length, 1.0 cm in width and 0.28 cm in height) on each side of the membrane. The effective membrane area was 2.0 cm^2^. All measurements were performed at room temperature using a volumetric flow rate of 0.2 L/min in the flow channels without any spacer. The FO performance of each membrane was evaluated in two different orientations: AL-FS or AL-DS with 2L DI water as the feed solution and 2L 2M NaCl solution as the draw solution. The FO performance of the membranes was evaluated by the water flux (Jv, Lm^−2^h^−1^, abbreviated as LMH) and reverse salt flux (Js, gm^−2^h^−1^, abbreviated as gMH) calculated from the following equations.


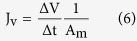



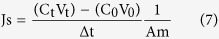


where ∆V (L) is the volume of water permeated across the FO membrane over a predetermined time ∆t (h) during the FO test, A_m_ is the effective membrane surface area (m^2^). C_t_ and V_t_ are the salt concentration (g/L) and the volume of the feed (L) at the end of the FO test respectively (their corresponding values at the start of the experiment are C_0_ and V_0_). Salt concentration was also measured by the conductivity meter. The dilution of the draw solution in the experiments was negligible since the ratio of water permeation flux to the volume of the draw solution was less than 1% during each filtration period.

During the FO long term performance test, AL-DS orientation was used with 100ml DI water as the feed solution and 2M NaCl solution as draw solution. The feed solution was replaced with DI water, and the draw solution was recovered to 2M for each 12 hour. The releases of Ca ions from the CaCO_3_ coated membranes to the feed solution were assessed via ICP-OES (opertima 7300DV, perkin elmer, USA). The solution was collected each 12 hour and acidified by 1% HNO_3_ prior analyzation.

## Additional Information

**How to cite this article**: Liu, Q. *et al.* Hydrophilic Mineral Coating of Membrane Substrate for Reducing Internal Concentration Polarization (ICP) in Forward Osmosis. *Sci. Rep.* **6**, 19593; doi: 10.1038/srep19593 (2016).

## Supplementary Material

Supplementary Information

## Figures and Tables

**Table 1:** Comparison of S parameter and FO performance of various FO membranes.

Membrane	S parameter (μm)	Water flux Jv (AL-FS/AL-DS) (LMH)	Reverse salt flux Js (AL-FS/AL-DS) (gMH)	Draw solution	Ref.
PES/PAA5/CaCO_3_	35.7	52/62	16.8/21.6	2M NaCl	This work
TFC membrane on cellulose ester substrate (with SDS/ glycerol and heat post treatment)	31.9	80.1/128.8	10.0/19.4	2M NaCl	(Ong, Chung *et al.* 2015)
TFC membrane on TiO_2_ incorporated polysulfone(Psf) substrate	390	33.0/59.4	15.7/31	2M NaCl	(Emadzadeh, Lau *et al.* 2014)
TFC membrane on carboxylated Psf substrate^*^	85*	18/27*	2/6*	1M MgCl2	(Cho, Han *et al.* 2013)
TFC membrane on zeolite incorporated Psf substrate^*^	340	40/86*	28/56*	2M NaCl	(Ma, Wei *et al.* 2013)
TFC membrane on SPEK blended Psf substrates	107	35/50	9/7	2M NaCl	(Han, Chung *et al.* 2012)
TFC membrane on PES/ SPSf-alloyed substrate	238	26.0/47.5	8.3/12.4	2M NaCl	(Wang, Chung *et al.* 2012)
TFC membranes on PESU-co-sPPSU/PESU(50 wt% sulfonation) substrate	324	21/33	2.2/2.8	2M NaCl	(Widjojo, Chung *et al.* 2011)
TFC membranes on polydopamine modified PSf substrate	1510	7.5/24	1.4/1.7	2M NaCl	(Han, Zhang *et al.* 2012)
HTI TFC membrane(prewetted)	620	15/32.5	7/18	1M NaCl	(Ren and McCutcheon 2014)

All FO tests were conducted using DI water as the feed solution, and all FO membranes in the Table were flat sheet membranes.

* Data estimated from graphs.

**Table 2:** Compositions of different membranes.

Dope 1: PES/PEG/NMP/Water = 20/37.9/37.9/4.2(w/w)		
Dope 2: PAA/NMP = 5/95(w/w)		
	Dope 1/vol.%	Dope 2/vol.%
PES/PAA0	100	0
PES/PAA3	97	3
PES/PAA5	95	5
PES/PAA10	90	10
