# Supplementary material for: Hydrophilic Mineral Coating of Membrane Substrate for Reducing Internal Concentration Polarization (ICP) in Forward Osmosis
Source: Sci Rep. 2016 Jan 22;6:19593. doi: 10.1038/srep19593 (PMC4726141; doi:10.1038/srep19593)
Supplement: Supplementary Information [file srep19593-s1.pdf]

Supporting information for

Hydrophilic Mineral Coating of Membrane Substrate for Reducing Internal Concentration Polarization (ICP) in Forward Osmosis

Qing Liu[+], Jingguo Li[+], Zhengzhong Zhou, Jianping Xie*, Jim Yang Lee*

Department of Chemical & Biomolecular Engineering,

National University of Singapore

10 Kent Ridge Crescent, Singapore 119260

* Corresponding author

Email: cheleejy@nus.edu.sg

Email: chexiej@nus.edu.sg

Fax: (65)-67791936

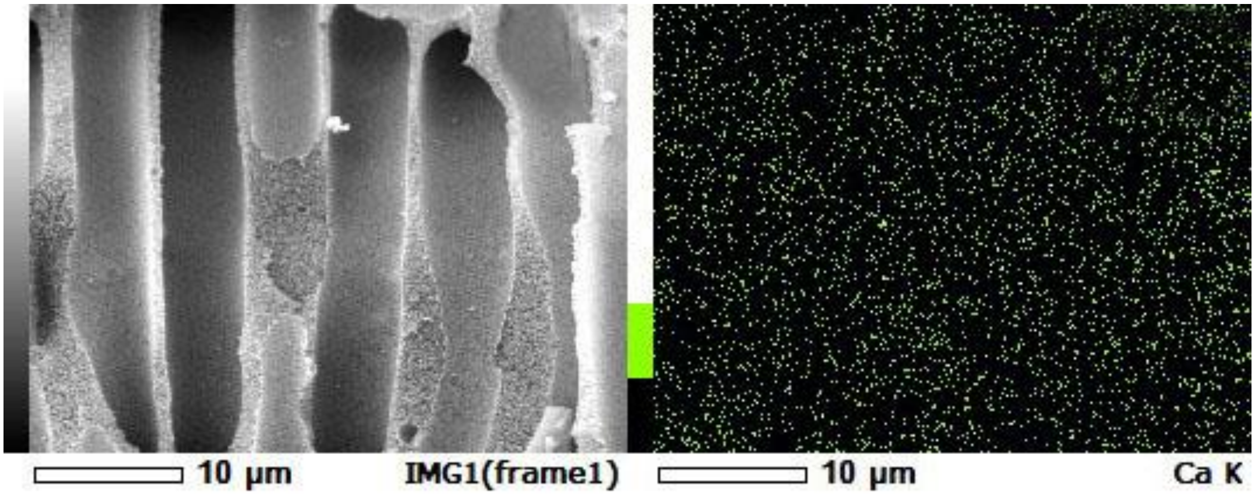

Figure S1: EDX result of the cross-section of PES/PAA5/$CaCO_3$ membrane

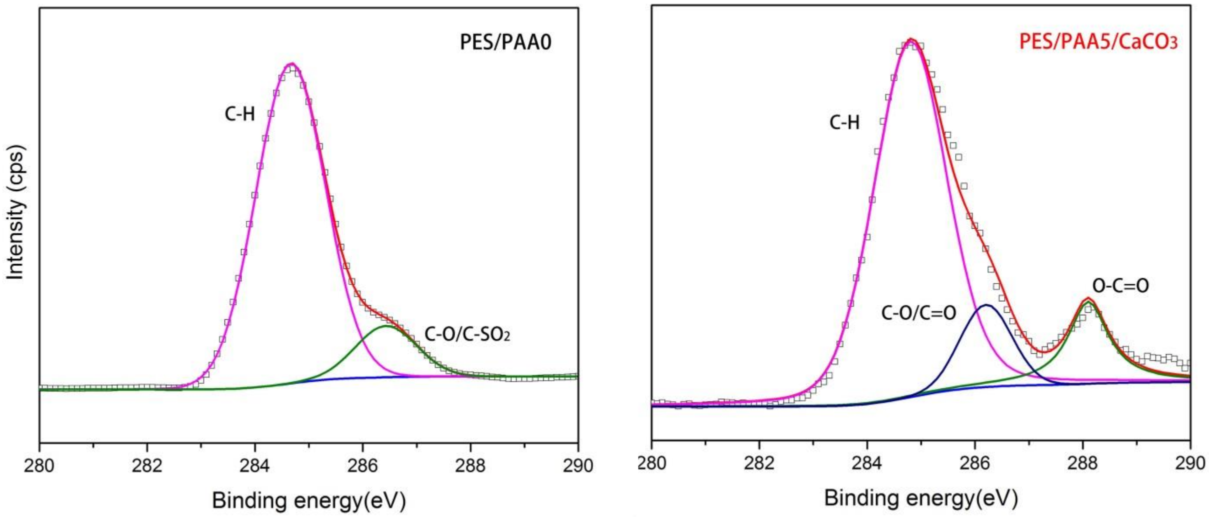

Figure S2: C 1s core level spectra of membranes

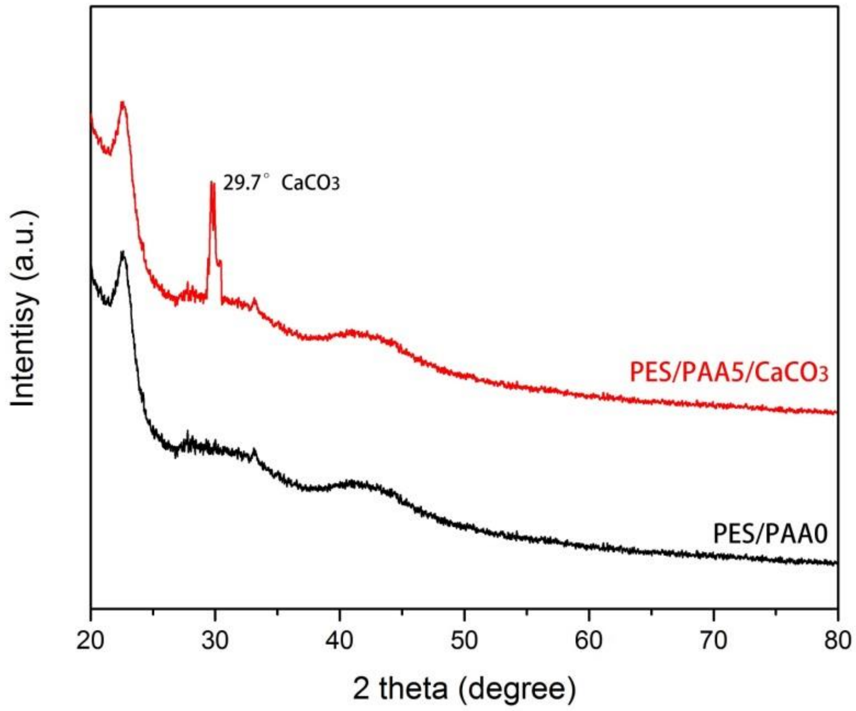

Figure S3: XRD patterns of PES/PAA0 membrane and PES/PAA5/$CaCO_3$ membrane.

Membrane	Young's modulus (MPa)	Tensile strength (MPa)	Elongation at break (%)
PES	152.0 ± 22.4	4.28 ± 0.6	17.1 ± 4.3
PES/PAA5	89.2 ± 11.6	3.69 ± 0.4	23.1 ± 5.2
PES/PAA5/$CaCO_3$	246.1± 35.6	5.74 ± 0.7	3.77 ± 2.6

Table S1: Mechanical properties of membranes

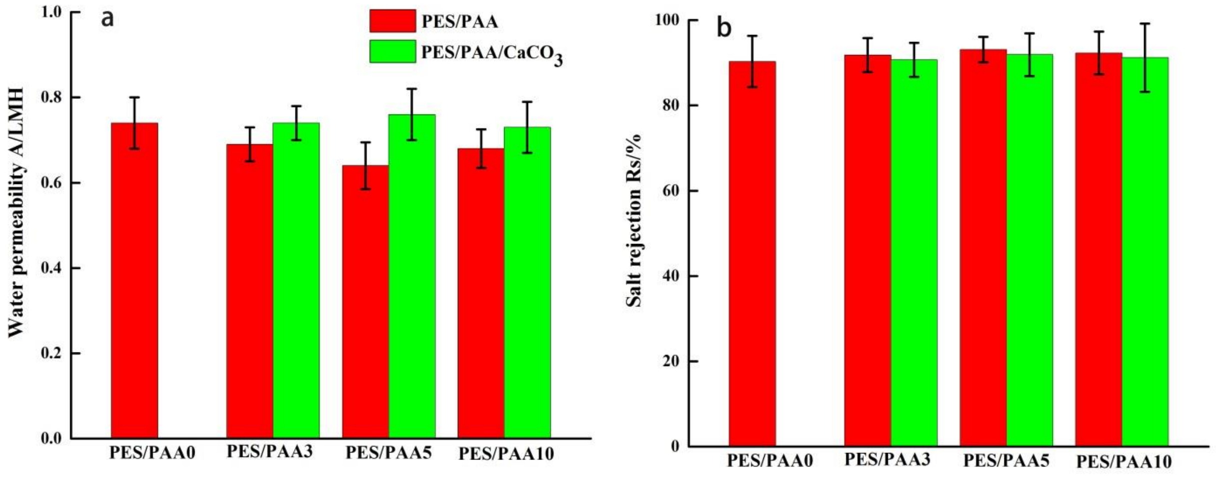

Figure S4: Intrinsic transport parameters (water permeability (A) and the salt (NaCl) rejection (R)) of membranes

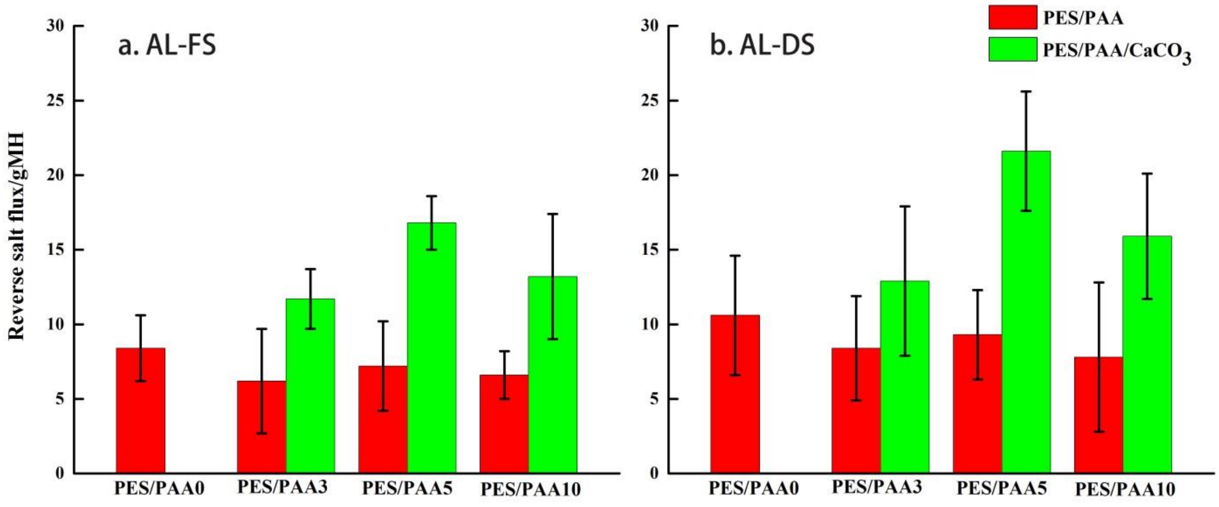

Figure S5: FO reverse salt flux of membranes at (a) AL-FS orientation and (b) AL-DS orientation

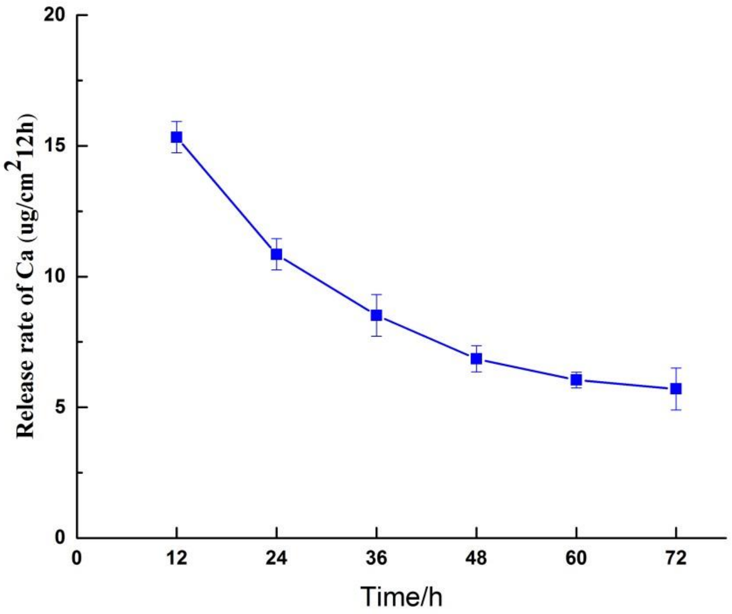

Figure S6: Calcium releasing rate of the PES/PAA5/$CaCO_3$ membrane.

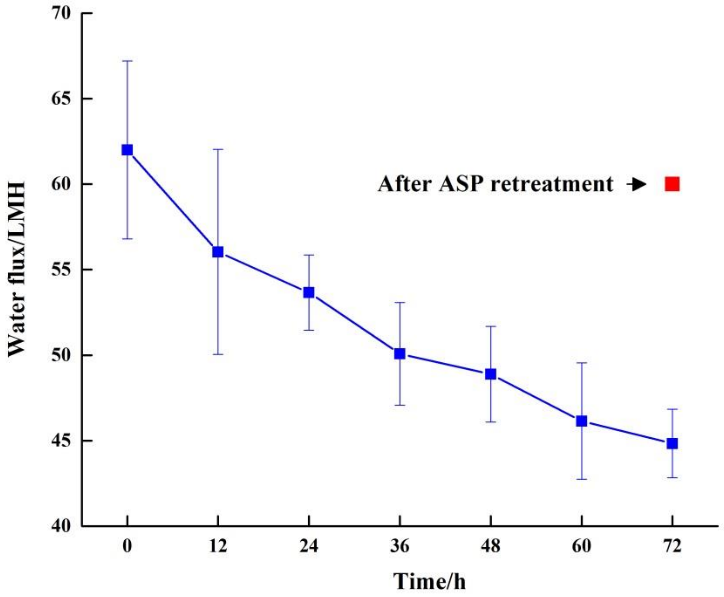

Figure S7: FO water flux decline in AL-DS mode with time
